# Knowledge and attitudes toward evidence-based cariology and restorative dentistry among Egyptian dental practitioners: a cross-sectional survey

**DOI:** 10.1186/s12903-023-03333-z

**Published:** 2023-09-01

**Authors:** Dina M. Elkady, Ahmad G. A. Khater

**Affiliations:** 1 Conservative Department, Faculty of Dentistry, Cairo University, Giza, Egypt; 2 Health Affairs Directorate, Egyptian Ministry of Health and Population, Banisuif, 62511 Egypt; 3 Faculty of Oral and Dental Medicine, Ahram Canadian University, Giza, Egypt

**Keywords:** Attitudes, Barriers, Dental care, Dental caries, Dental practice, Evidence-based dentistry, Questionnaire survey, Restorative dentistry

## Abstract

**Background:**

This is the first study to assess Egyptian dental practitioners’ knowledge about conservative caries management approaches and investigate whether this knowledge transfers into clinical practice and the barriers to translating research into evidence-based practice.

**Methods:**

A sample of dental practitioners was surveyed using an online questionnaire. Convenience and snowball sampling were used to collect data from February to June 2022. We included graduated dentists from Egyptian universities who practiced in Egypt. Data were analyzed with descriptive statistics, and the associations between variables were checked using Kruskal Wallis and Chi-Square tests.

**Results:**

This study included 396 participants from throughout Egypt. There were significant correlations between specialty and participants’ knowledge and behaviors toward evidence-based caries management (*p* = 0.002) and between specialization and tools used to detect carious lesions (*p* < 0.001). Most participants (59.1%) used G.V Black’s classification, and (80.8%) removed caries based on the feature of dentin hardness and color, whereas (67%) removed caries until hard dentine remained. The participants’ primary hurdle to staying up-to-date was their belief that the newly gained information would not be clinically applicable due to a lack of equipment or working in low-economic areas. Patient-related barriers were the major obstacles for participants in implementing evidence-based practice.

**Conclusion:**

Egyptian dentists did not fully embrace minimal invasive approaches for caries management, and practitioners’ experiences continue to shape decision-making. It emphasizes the imperative to practically educate dentists using effective knowledge translation dissemination to promote evidence adoption in daily practice and advocate value-based dental care to address the economic crisis’s impact on Egypt’s healthcare.

**Supplementary Information:**

The online version contains supplementary material available at 10.1186/s12903-023-03333-z.

## Introduction

Dental caries remains a significant health problem worldwide [1], affecting 60–90% of children and the majority of adults [2]. There are health inequalities in dental caries burden in both children and adults [3], particularly most prevalent in low-income communities due to unaffordable dental care [4, 5]. As such, 2.4 billion people (35% global prevalence) had carious permanent teeth without treatment, making it the most common medical condition [6]. Hence, dental caries poses a global health burden for numerous people who require proper treatment to improve their quality of life, particularly those who cannot afford dental care [3, 7, 8]. Similarly, dental caries remains a crucial health concern in Egypt, particularly among vulnerable populations (e.g., women and persons with low socioeconomic status), with approximately 70.3% having at least one untreated carious tooth [9].

Caries-management concepts have shifted substantially in recent years towards a deeper understanding of caries’ pathophysiology and tissue healing capabilities, resulting in more conservative approaches [10, 11]. Minimal intervention dentistry was developed to address the deficiencies in the traditional operative concept (“drill and fill”) by providing a cause-based and comprehensive caries management philosophy that includes early caries detection, new caries lesions prevention, and management of carious lesions through minimally invasive procedures [12, 13]. Such a recent approach aims to preserve the tooth as much as possible to boost its life span by conservatively restoring it, converting the concept of “extension for prevention” into “prevention of extension” [14]. However, caries management and restorative dentistry are becoming increasingly complex, putting clinical decision-making under increased strain due to innovations in techniques and materials, socio-demographic shifts, more knowledgeable patients, and abundantly available research [15].

In the last decades, the evidence-based practice concept has emerged in dentistry in responding to the imperative to improve the quality of standard patient care [16–18], which was recognized as making clinical decisions based on the integration of the best research evidence available with clinician expertise and patient values [19]. In other words, an approach that assists healthcare practitioners in offering the best possible care to patients by integrating the best research with clinical practice. However, the responsibility of looking for, appraising critically, and interpreting research findings in daily practice should not be restricted solely to clinicians [20]. In this regard, many organizations such as the International Caries Consensus Collaboration (ICCC), the American Dental Association (ADA), and the European Dental Association (EDA) have worked to develop recommendations for minimally invasive caries management, intending to improve dental care and maintain teeth healthy and functional for life [21–24].

Still, there is a big gap between research evidence and dental practice that leads to the loss of the evidence-based practice value in patient dental care [25, 26], reflecting that many dentists still prefer to rely mostly on knowledge learned via education or personal experience, and many still need to embrace the concept of minimal intervention dentistry completely [27, 28]. Therefore, we aim to assess the knowledge of Egyptian dental practitioners about conservative caries management approaches and investigate whether this knowledge transfers into clinical practice and the barriers to translating research evidence into evidence-based practice.

## Methods

### Study design

This study followed the STROBE guidelines for reporting cross-sectional observational studies [29]. The study protocol was a priori registered in the Open Science Framework and approved by the Research Ethics Committee, Faculty of Dentistry, Cairo University, Egypt [30]. An online questionnaire was employed, and the survey was conducted during the spring of 2022. Participation was voluntary, and there were no rewards or penalties for participating.

### Questionnaire

The questionnaire included several question styles (yes/no questions, multiple choice questions, open-ended questions, and statements with Likert scale responses). After reviewing published cariology surveys, we designed these questions to assess three primary areas: knowledge (including evidence-based caries diagnosis and management according to the recent ICCC and ADA consensus [21, 31, 32], continuing education, and evidence-based practice (including enablers and barriers). We assessed the content validity of the initial questionnaire in person using a paper-based method, considering the differences in professions and education to guarantee that people from varied educational backgrounds would easily understand it. Intra-rater reliability was assessed on this pilot sample of ten participants who were academics, residents, and dentists, with one month between the test and retest. We used the Spearman correlation to test the correlation between the two rounds, and questions with low reliability were removed. The final questionnaire was transferred into a Google Form and distributed.

The questionnaire started with an overview of the study objectives and target population, highlighting that participation was voluntary. The subsequent questions focused on four areas (Supplementary file):

- Demographic data (gender, graduation year, university, specialty, etc.)
- Questions evaluating participants’ knowledge and behavior toward caries management in terms of evidence-based practice.
- Questions about participants’ behavior toward continuing education and keeping up-to-date on cariology and restorative dentistry, as well as the barriers to doing so.
- Questions about participants’ attitudes towards evidence-based dentistry and their barriers to establishing an evidence-based practice.

### Setting and participants

Convenience and snowball sampling were performed. We contacted university course directors, inviting them to complete and distribute the questionnaire to their residents, intern, and postgraduate students. Also, we used social media platforms to reach general practitioners and cover dental professionals throughout Egypt. The survey was opened in February and closed in June of 2022. We included graduated dentists with a bachelor’s or higher degree from Egyptian universities who practiced in Egypt. We excluded undergraduate students and dentists who obtained their undergraduate qualification abroad or practiced outside Egypt.

### Sample size

While World Health Organization (WHO) estimated the number of dentists in Egypt to be 32,848 in December 2018 [33], the Egyptian Dental Syndicate statistics assume this number to be 76,843 at present. As a result, we calculated our sample to represent a total population of 76,843 with a 95% confidence level and a 5% margin of error, resulting in a minimum sample size of 382. Sample size calculation was performed using Epi Info™ 7.2 (Centers for Disease Control and Prevention (CDC), Atlanta, USA) [34].

### Statistical analysis

Descriptive statistics were used to present the demographic characteristics of the participants and their questionnaire responses as frequencies and percentages for categorical variables. Kruskal Wallis and Chi-Square tests were performed for group comparisons according to the respective data. Statistically significant differences were assumed if *p* < 0.05. All statistical analyses were performed using SPSS 28 (IBM. Armonk, USA).

## Results

### Participants’ demographic characteristics

Due to the nature of our recruitment methods (respondent-driven sampling), we could not estimate the total number of dental professionals who received our survey invitation. Among the 410 questionnaires received, 14 were excluded because they did not meet the eligibility criteria; ten obtained their bachelor’s degree abroad, three were duplicated, and one had incomplete responses. After excluding these invalid responses, we included 396 individuals from throughout Egypt (44.9% men and 63.1% women), with 44.9% being practicing dentists, 20.5% intern students, 16.4% academics (i.e., non-clinical specialties), 13.6% postgraduate students, and 4.5% residents. The majority (71.5%) graduated from public universities; 28.5% graduated from private universities. 49.2% were general practitioners, 17.7% restorative dentistry specialists, and 12.6% specialized in oral and maxillofacial surgery or prosthodontics. Most participants (82.6%) worked in capitals, with 8.3% in the delta region, 6.1% in upper Egypt, and 1.5% in the east. 53.5% worked in the public sector and 46.5% in the private sector. 65.2% of participants had a BDS degree, 34.8% a postgraduate degree (52.2% MDS, 29.7% Ph.D., and 14.5% specialized diploma). (Table 1)

**Table 1:** Demographic characteristics of the participants (N = 396)

		N	%
Gender	Female	250	63.1
	Male	146	36.9
Profession	Dentist	178	44.9
	Intern student	81	20.5
	Academic	65	16.4
	Postgraduate student	54	13.6
	Current Resident	18	4.5
From which university did you graduate?	Public university	283	71.5
	Private university	113	28.5
Specialty	General Dentistry	195	49.2
	Restorative dentistry	70	17.7
	Oral and Maxillofacial Surgery and Prosthodontics	50	12.6
	Orthodontics and Pediatric Dentistry	33	8.3
	Endodontics	27	6.8
	Oral Medicine, Pathology, and Radiology	9	2.3
	Periodontics	7	1.8
	Academic specialty	5	1.3
Postgraduate study	Yes	138	34.8
	No	258	65.2
Highest degree	MDS	72	52.2
	Ph.D.	41	29.7
	Specialized Diploma	20	14.5
	Membership in Orthodontics (MOrtho)	4	2.9
	Diploma of Membership of the Faculty of Dental Surgery (MFDS)	1	0.7
University of your highest degree?	Public university	127	92
	Private university	5	3.6
	International university	6	4.3
Primary sector of practice	Public	212	53.5
	Private	184	46.5
Location of practice	Capitals	327	82.6
	Delta area	33	8.3
	Upper Egypt	24	6.1
	East area	6	1.5
	North area	6	1.5

### Knowledge towards caries management and restorative dentistry

Table 2 presents the results of the participants’ responses to questions about knowledge and behavior toward evidence-based cariology and restorative dentistry. There was a significant relationship between specialty and total score of the questions evaluating participants’ knowledge and behavior toward caries management in terms of evidence-based practice (*p* = 0.002), with restorative dentists having a higher median score (median = 16, IQR = 4.25) than oral medicine, pathology, and radiology specialists (median = 12, IQR = 3), oral and maxillofacial surgery and prosthodontics specialists (median = 14, IQR = 3.25), and general practitioners (median = 14, IQR = 4) (Appendix Table 1). However, there was a significant correlation between specialization and tools used to detect carious lesions (*p* < 0.001) since oral medicine, pathology, and radiology dentists (88.9%) used visual and tactile examination to detect carious lesions more than other disciplines, while academics (60.0%) used visual-tactile and radiographic examination. Additionally, the percentage of dentists who used caries-detector dyes to detect carious lesions was higher in endodontists (11.1%) than in other specialties; also, those who used recent diagnostic tools to detect carious lesions were higher in endodontists (7.4%) (Appendix Table 2).

**Table 2:** Participants’ responses to questions about caries management and restorative dentistry

			N	%
What is your preferred caries classification for application in your dental practice?	G.V. Black’s classification		234	59.1
	ICDAS classification		55	13.9
	Mount & Hume (Si/Sta) classification		39	9.8
	American Dental Association (ADA) Caries Classification		10	2.5
	WHO system classification		9	2.3
	Classifying according to position (Occlusal, Proximal, and Cervical)		5	1.3
	Classifying according to severity (Mild, Moderate, Sever)		2	0.5
	Miller classification		1	0.3
	Irrelevant answer		17	4.3
	No answer		24	6.1
What tools do you use to detect a carious lesion?	Visual and tactile examination		180	45.5
	Visual-tactile and radiographic examination		134	33.8
	Caries-Detector dyes		29	7.3
	Dental excavator		18	4.5
	Radiographic examination		15	3.8
	Recent diagnostic tools		13	3.3
	Irrelevant answer		7	1.8
Management of caries lesion depends on its activity		Strongly agree	109	27.5
		Agree	235	59.3
		Unsure	21	5.3
		Disagree	29	7.3
		Strongly disagree	2	0.5
A cavity-free patient indicates that he/she is caries-free		Strongly agree	16	4
		Agree	52	13.1
		Unsure	69	17.4
		Disagree	173	43.7
		Strongly disagree	86	21.7
The new approach of caries management depends on the ability of dental tissue remineralization		Strongly agree	113	28.5
		Agree	201	50.8
		Unsure	63	15.9
		Disagree	19	4.8
		Strongly disagree	0	0
The new concept of caries management is based on the sealing of cavities by altering the ecological niche of cariogenic bacteria		Strongly agree	88	22.2
		Agree	195	49.2
		Unsure	91	23
		Disagree	21	5.3
		Strongly disagree	1	0.3
Dentin hardness and color are the most critical features of dentin during cavity preparation		Strongly agree	79	19.9
		Agree	241	60.9
		Unsure	45	11.4
		Disagree	25	6.3
		Strongly disagree	6	1.5
Resistance to excavation is a characteristic sign during caries management		Strongly agree	75	18.9
		Agree	239	60.4
		Unsure	49	12.4
		Disagree	27	6.8
		Strongly disagree	6	1.5
It is necessary to remove soft dentin from the wall cavity		Strongly agree	207	52.3
		Agree	138	34.8
		Unsure	23	5.8
		Disagree	24	6.1
		Strongly disagree	4	1
Complete caries removal to hard dentin is the best treatment option for small to moderate-sized cavities		Strongly agree	104	26.3
		Agree	161	40.7
		Unsure	49	12.4
		Disagree	61	15.4
		Strongly disagree	21	5.3
The step-wise caries excavation is the best treatment for a deep lesion		Strongly agree	55	13.9
		Agree	196	49.5
		Unsure	71	17.9
		Disagree	62	15.7
		Strongly disagree	12	3
Step-wise caries removal is a type of selective caries removal		Strongly agree	78	19.7
		Agree	248	62.6
		Unsure	48	12.1
		Disagree	17	4.3
		Strongly disagree	5	1.3
In case of a cavitated lesion, a minimally invasive approach could be an alternative treatment		Strongly agree	54	13.6
		Agree	155	39.1
		Unsure	93	23.5
		Disagree	77	19.4
		Strongly disagree	17	4.3
Caries diagnosis depends on dentin stains and cavitation		Strongly agree	22	5.6
		Agree	147	37.1
		Unsure	63	15.9
		Disagree	122	30.8
		Strongly disagree	42	10.6
The risk assessment of the patient influences the management of caries		Strongly agree	187	47.2
		Agree	181	45.7
		Unsure	21	5.3
		Disagree	7	1.8
		Strongly disagree	0	0
The history of pain is critical in the caries management		Strongly agree	163	41.2
		Agree	194	49
		Unsure	24	6.1
		Disagree	13	3.3
		Strongly disagree	2	0.5
Routine x-rays on every patient are essential for caries detection		Strongly agree	71	17.9
		Agree	126	31.8
		Unsure	58	14.6
		Disagree	114	28.8
		Strongly disagree	27	6.8
Periapical radiographs are more effective than Bitewing in caries diagnosis		Strongly agree	21	5.3
		Agree	41	10.4
		Unsure	47	11.9
		Disagree	176	44.4
		Strongly disagree	111	28
Pulp testing may not be essential during caries management		Strongly agree	28	7.1
		Agree	133	33.6
		Unsure	72	18.2
		Disagree	132	33.3
		Strongly disagree	31	7.8
The explorer probe is necessary for caries diagnosis		Strongly agree	123	31.1
		Agree	197	49.7
		Unsure	25	6.3
		Disagree	40	10.1
		Strongly disagree	11	2.8
ICDAS classification is more accurate than Si/Sta classification		Strongly agree	55	13.9
		Agree	118	29.8
		Unsure	180	45.5
		Disagree	35	8.8
		Strongly disagree	8	2
Caries care, Caries management, and Caries control all have the same meaning		Strongly agree	24	6.1
		Agree	80	20.2
		Unsure	63	15.9
		Disagree	162	40.9
		Strongly disagree	67	16.9
The non-invasive approach is only indicated for patients with a high caries risk		Strongly agree	13	3.3
		Agree	28	7.1
		Unsure	80	20.2
		Disagree	166	41.9
		Strongly disagree	109	27.5
The cavity sealing approach is only used on the intact enamel surface		Strongly agree	46	11.6
		Agree	137	34.6
		Unsure	125	31.6
		Disagree	71	17.9
		Strongly disagree	17	4.3
Sealing of the cavity could remineralize it		Strongly agree	50	12.6
		Agree	172	43.4
		Unsure	93	23.5
		Disagree	69	17.4
		Strongly disagree	12	3
It is necessary to seal inactive root caries		Strongly agree	47	11.9
		Agree	167	42.2
		Unsure	126	31.8
		Disagree	49	12.4
		Strongly disagree	7	1.8

### Behaviors toward continuing education

Most participants agreed that there had been a significant change in the operative dental practice since graduation (78.8%), and 26.5% preferred the in-person courses and workshops to stay up-to-date with cariology and restorative dentistry (Table 3). However, there was a significant correlation between postgraduate education and the belief that there has been a significant change in the operative dental practice from graduation year (*p* < 0.001) since the dentists who believed that and pursued postgraduate education were 94.9%. Also, there was a significant correlation between that belief and years since graduation (*p* < 0.001), as 96.4% of dentists who believed that there had been a significant change in the operative dental practice had 10 to 14 years following graduation (96.4%) (Appendix Table 3). The main barrier to updating their cariology and restorative dentistry knowledge for 62.7% was that newly gained information would not be clinically applicable due to lack of equipment or working in a low-income area, while 58.3% of them stated the high cost of continuing education courses was the barrier. The American Dental Association (34.7%) was the top organization where participants usually followed its recent consensus and guidelines in cariology and restorative dentistry, followed by university curriculums (25.5%).

**Table 3:** Participants’ responses to questions about continuing education in cariology and restorative dentistry

		N	%
Do you believe there has been a significant change in the operative dental practice since your graduation year?	No	25	6.3
	Maybe	59	14.9
	Yes	312	78.8
What is your preferred method of staying up-to-date with cariology and restorative dentistry?	In-person courses and workshops	105	26.5
	Journal articles	84	21.2
	University programs and degrees	74	18.7
	Online courses	68	17.2
	Conferences	47	11.9
	Textbooks	14	3.5
	None	2	0.5
	Asking friends	1	0.3
	Discussions	1	0.3
How many continuing education courses in cariology and restorative dentistry did you take in the last year?	No	193	48.7
	Yes	203	51.3
How many international scientific articles on cariology, and restorative dentistry did you read in the last year?	No	151	38.1
	Yes	245	61.9
What do you feel are the barriers to updating your cariology and restorative dentistry knowledge?	The newly gained information will not be clinically applicable due to lack of equipment or working in the low-economic area.	248	62.7
	Courses for continuous education are expensive.	231	58.3
	I think there has been no significant development in the field since my graduating year.	34	8.7
	No barriers	9	2.3
	Cariology and restorative dentistry are outside my scope	8	2
	No time available for continuing education	7	1.8
	Unable to find trusted information	3	0.8
	Lack of motivation	2	0.6
	Patients awareness	1	0.3
Do you always follow the recent consensus and guidelines in cariology and restorative dentistry?	No	91	23
	Maybe	186	47
	Yes	119	30.1
If yes, whose organizations or committees do you adhere to their consensus and guidelines?	American Dental Association (ADA)	34	34.7
	University curriculum	25	25.5
	Irrelevant answer	11	11.2
	Expert opinions	7	7.1
	Royal College of Surgeons in Ireland (RCSI)	4	4.1
	British Dental Association (BDA)	3	3.1
	Didn’t know	3	3.1
	European Organization for Caries Research (ORCA)	3	3.1
	World Health Organization (WHO)	2	2
	FDI World Dental Federation	2	2
	American Academy of Cosmetic Dentistry (AACD)	1	1
	American Academy of Pediatric Dentistry (AAPD)	1	1
	International Caries Consensus Collaboration (ICCC)	1	1
	NICE guidelines	1	1

### Attitudes toward the evidence-based dental practice

Most participants agreed that it is difficult to find a trusted source for evidence-based information (41.9%) and hard to understand the results of scientific articles due to the statistical portion (38.9%). They also agreed that the evidence-based information is time-consuming, not clinically applicable in daily practice (39.6%), and not clinically applicable due to the economic burden (44.7%). The main barrier to establishing an evidence-based practice in cariology and restorative dentistry for 32.1% of participants was the financial barrier, followed by the inability of participants to find trusted information (16.9%) (Table 4). However, there was a significant correlation between getting a trusted source for evidence-based information and postgraduate education (*p* < 0.001), as the participants who did not pursue postgraduate education and found it difficult were 43.4%; in contrast, those who disagreed and took a postgraduate education were 47.1%. Furthermore, there was a significant correlation between postgraduate education and the feeling that some evidence-based information is time-consuming and not clinically applicable in daily practice (*p* = 0.001) since the participants who agreed with that and did not pursue postgraduate education were 45.3%; in contrast, participants who disagreed and took a postgraduate education were 30.4% (and 8.7% strongly disagreed) (Appendix Table 3).

**Table 4:** Participants’ responses to questions about their attitudes towards evidence-based dentistry

			N	%
I find it hard to get a trusted source for evidence-based information	Strongly agree		40	10.1
	Agree		166	41.9
	Unsure		68	17.2
	Disagree		114	28.8
	Strongly disagree		8	2
I find it hard to understand the results of scientific articles due to the statistical portion	Strongly agree		40	10.1
	Agree		154	38.9
	Unsure		62	15.7
	Disagree		136	34.3
	Strongly disagree		4	1
I find it hard to understand the guidelines and consensus	Strongly agree		13	3.3
	Agree		85	21.5
	Unsure		97	24.5
	Disagree		180	45.5
	Strongly disagree		21	5.3
I feel some evidence-based information is time-consuming and not clinically applicable in my daily practice	Strongly agree		39	9.8
	Agree		157	39.6
	Unsure		80	20.2
	Disagree		102	25.8
	Strongly disagree		18	4.5
I feel the evidence-based information is not applicable in daily practice due to the economic burden	Strongly agree		61	15.4
	Agree		177	44.7
	Unsure		75	18.9
	Disagree		69	17.4
	Strongly disagree		14	3.5
What is the main challenging aspect of performing evidence-based cariology and restorative dentistry?	Economical barrier		127	32.1
	Unable to find trusted information		67	16.9
	Patient awareness		47	11.9
	Not clinically applicable		41	10.4
	Irrelevant answer		40	10.1
	Lack of equipment		29	7.3
	Time consuming		27	6.8
	Low socioeconomic level of patients		24	6.1
	Didn’t know		20	5.1
	No answer		19	4.8
	Teaching methods of evidence-based dentistry		15	3.8
	No challenges		7	1.8

## Discussion

With the emergence of conservative and less invasive caries management approaches, it is crucial to assess dental practitioners’ knowledge and attitudes regarding these strategies and how research can improve the quality of dental care. In this regard, this is the first study to assess Egyptian dental professionals’ knowledge of current caries management approaches and investigate the barriers to translating this knowledge into daily clinical practice.

Dental caries is a dynamic process with a recurring disease cycle involving demineralization and remineralization over time [35]; considering this modern concept in diagnosis and risk assessment are key underpins for successfully understanding and controlling dental caries. In this study, most dentists (59.1%) used G.V Black’s classification in their dental practice, which means that their caries management was based solely on caries location without considering caries activity or lesion depth, potentially resulting in more invasive treatment [36]. Although several systems were used to classify dental caries, the International Caries Detection and Assessment System (ICDAS) has evolved to be more comprehensive and appropriate to current minimally invasive approaches, and current guidelines recommend it in clinical practice [22, 37]. This system classifies caries based on visual signs of the lesion severity considering sound surfaces and caries phases and combining these findings with radiographic data; hence, it aids in early caries diagnosis and assessment of complete caries prevalence [38–40].

Most dentists (about 80.8%) removed caries based on the feature of dentin hardness and color, and (about 67%) removed caries in small to moderate-sized cavities until hard dentine remained. Such behavior relies on the correlation between bacterial count and dentin hardness [41, 42]; hence, removing caries until the remaining dentin becomes hard reduces residual bacteria at the cavity floor. Caries removal solely based on dentin characteristics regardless of the caries condition (i.e., active or arrest) results in over-treatments, removal of many arrested lesions, and complications if softened dentin is near the pulp [43, 44].

The results revealed that most practitioners (more than 52.3%) still relied on the conventional approach of removing all soft dentine and considered step-wise excavation the best treatment for deep lesions (around 63.4%). Such an approach raises the risk of pulp exposure and issues; pulp exposure treatment is costly and may compromise tooth longevity; still, it is either invasive or unsuccessful in the long term [45–47]. However, dentists continued to use this approach due to the incorrect belief that residual carious dentine or bacteria in the cavity could damage the pulp or cause caries progression [48–50]. Moreover, the belief that step-wise excavation is the best alternative to the complete excavation concept contradicts current evidence due to the high cost-benefit ratio and high risk of pulp exposure during the second visit [51, 52].

Without a doubt, the main goal of healthcare practitioners is to provide patients with the best possible care. Although several studies assessed dental students’ and practitioners’ understanding of conservative caries management approaches, they were focused mainly on the knowledge and its implication on daily practice [53–56]. However, patient care will not improve unless this theoretical knowledge is applied in daily dental practice. Therefore, the real challenge in improving healthcare quality and implementing evidence-based approaches is not in evidence dissemination or knowledge but in translating this knowledge from research into evidence-based dental practice. As such, recognizing the obstacles is the first step toward implementing a dental practice based on research evidence, which includes hurdles related to the practitioner, evidence, context, and patient [57]. 23% of participants did not follow the caries management guidelines in their practices. Such behavior was primarily due to evidence-related barriers since 52% found it hard to obtain a trusted source, and 50% found it hard to understand the scientific articles’ results due to the statistical aspect. Such a barrier is highly evident by the unmanageable amount of existing research (research waste); for example, there were 104,975 dental articles published between 2009 and 2019 on PubMed [58], making it impossible for dental practitioners to read, understand, and incorporate all this research into daily practice. Hence, resources that provide summarized intervention effectiveness reviews are crucial for promoting evidence-based practice. Another barrier is how results are often presented, emphasizing the significance of including a plain language summary with research articles. Moreover, it highlights the imperative of investigating the effectiveness of other means of research knowledge translation (e.g., videos, toolkits, and social media) rather than written materials in promoting evidence uptake in daily dental practice [59].

The main barrier for 62.7% of participants to update their knowledge was that the newly gained information would not be clinically applicable due to a lack of equipment or working in a low-economic area. This misconception stems from the common belief that evidence-based practice is synonymous with more scientific and expensive practice, contrary to the purpose of evidence-based practice: patient-centered and empirically grounded care to provide optimal clinical decisions [57, 60]. Such beliefs represent an evident gap between researchers’ and practitioners’ communities, as practitioners are unable to comprehend the complexity of the research process; on the other hand, researchers focus more on the quality of knowledge obtained from their studies, with little attention paid to the relevance of this knowledge for practitioners and patients, as well as the complexities of applying it in patient care [61, 62]. As a result, promoting evidence-based dental practice necessitates incorporating evidence assessment and synthesis skills in dental curriculum and training to ensure that the workforce will be filled with practitioners who can comprehend and use research evidence in their daily practices [63, 64]. Also, more extensive dissemination of easily accessible evidence reviews on clinical topics (e.g., scoping and systematic reviews and guidelines) can encourage interest in and promote applying research evidence in daily practice [65]. Moreover, such a gap between the communities of researchers and practitioners can only be bridged by incorporating practitioners in the research process and giving them voices in determining the challenges and dilemmas they encounter and underpin their profession [57, 66, 67].

Patient-related barriers (i.e., economic, awareness, and social levels) were the major obstacles for participants in implementing evidence-based practice. Such barriers are rational and supposed to increase globally due to the rise in living costs and economic crisis, often affecting the patient’s dental care and preferences [68, 69]. This emphasizes the crucial need to raise the value of dental research, prioritize future investigations, and advocate contextualized value-based dental care based on economic evaluation rather than effectiveness alone [70, 71]. In other words, funding and publishing priority should be given to research that addresses a real-world issue that matters to patients and clinicians, with attempts to eliminate the research waste and repetition [72, 73].

This study has some limitations; first, this research is based on quantitative evaluations by collecting data using an electronic survey; hence, it could not provide a deeper understanding of the participant’s knowledge and the barriers and enablers of implementing evidence-based dental practice in Egypt. Second, participants were recruited using non-probability sampling methods since this study was the first to be done about the cariology and evidence-based dentistry era in Egypt. Therefore, more population-based surveys and qualitative studies are needed to support these findings, provide a deeper grasp of the participants’ knowledge and barriers and enablers of the evidence-based dental practice, and further explain the reasons behind their behaviors.

## Conclusion

In Egypt dental practices, some conventional concepts (e.g., complete caries removal, using traditional caries classifications, removing caries based on dentin features and till hard dentine) remain prevalent, and practitioners’ experience and familiarity are still predominant in shaping clinical decision-making. Practitioners could not utilize research evidence in their dental practice due to some patient-related (e.g., rising living costs and high cost of dental care) and evidence-related (e.g., difficulty understanding research results and inaccurate beliefs) barriers. As a result, it emphasizes the imperative of practically teaching dental practitioners in minimal intervention approaches through effective research knowledge translation means rather than written materials and guidelines, as well as the prioritization of dental research and advocating value-based dental care to address the economic crisis’s impact on Egypt’s healthcare.

### Electronic supplementary material

Below is the link to the electronic supplementary material.

Supplementary Material 1

Supplementary Material 2

## Data Availability

The data that support the findings of this study are available from the corresponding author [A.G.A.K] upon reasonable request.
