# Supplementary material for: Knowledge and attitudes toward evidence-based cariology and restorative dentistry among Egyptian dental practitioners: a cross-sectional survey
Source: BMC Oral Health. 2023 Sep 1;23:622. doi: 10.1186/s12903-023-03333-z (PMC10474780; doi:10.1186/s12903-023-03333-z)
Supplement: Supplementary file 1 — Supplementary Material 1 [file 12903_2023_3333_MOESM1_ESM.docx]

**Appendix Table S1. Comparison of practice score across university, years since graduation, and specialty**

|  |  | Median | IQR | P-value |
| --- | --- | --- | --- | --- |
| University of graduation | Public University | 15.0 | 5.00 | 0.131 |
|  | Private University | 14.0 | 4.00 |  |
| Years since graduation | Less than 5 | 15.0 | 4.00 | 0.483 |
|  | 5 to 9 | 15.0 | 3.00 |  |
|  | 10 to 14 | 14.0 | 4.75 |  |
|  | 15 or more | 14.0 | 5.00 |  |
| Specialty | Academic specialty | 14.0 | 3.50 | **0.002** |
|  | Endodontic | 15.0 | 4.00 |  |
|  | General Dentistry | 14.0 | 4.00 |  |
|  | Oral and Maxillofacial Surgery and Prosthodontics | 14.0 | 3.25 |  |
|  | Oral Medicine, Pathology, and Radiology | 12.0 | 3.00 |  |
|  | Orthodontics and Pediatric Dentistry | 14.0 | 3.00 |  |
|  | Periodontics | 15.0 | 3.00 |  |
|  | Restorative dentistry | 16.0 | 4.25 |  |

|  | | | Tools to detect a carious lesion | | | | | | | P-value |
| --- | --- | --- | --- | --- | --- | --- | --- | --- | --- | --- |
|  |  |  | *Caries-Detector dyes* | *Dental excavator* | *Radiographic examination* | *Recent diagnostic tools* | *Visual and tactile examination* | *Visual-tactile and radiographic examination* | *Irrelevant*  *answer* |  |
| Specialty | Academic specialty | N | 0 | 0 | 0 | 0 | 2 | 3 | 0 | **<0.001** |
|  |  | % | 0.0% | 0.0% | 0.0% | 0.0% | 40.0% | 60.0% | 0.0% |  |
|  | Endodontic | N | 3 | 0 | 1 | 2 | 9 | 11 | 1 |  |
|  |  | % | 11.1% | 0.0% | 3.7% | 7.4% | 33.3% | 40.7% | 3.7% |  |
|  | General Dentistry | N | 20 | 13 | 10 | 4 | 86 | 58 | 4 |  |
|  |  | % | 10.3% | 6.7% | 5.1% | 2.1% | 44.1% | 29.7% | 2.1% |  |
|  | Oral and Maxillofacial Surgery and Prosthodontics | N | 1 | 1 | 1 | 2 | 19 | 26 | 0 |  |
|  |  | % | 2.0% | 2.0% | 2.0% | 4.0% | 38.0% | 52.0% | 0.0% |  |
|  | Oral Medicine, Pathology, and Radiology | N | 0 | 0 | 0 | 0 | 8 | 1 | 0 |  |
|  |  | % | 0.0% | 0.0% | 0.0% | 0.0% | 88.9% | 11.1% | 0.0% |  |
|  | Orthodontics and Pediatric Dentistry | N | 1 | 2 | 2 | 0 | 15 | 12 | 1 |  |
|  |  | % | 3.0% | 6.1% | 6.1% | 0.0% | 45.5% | 36.4% | 3.0% |  |
|  | Periodontics | N | 0 | 0 | 0 | 0 | 5 | 2 | 0 |  |
|  |  | % | 0.0% | 0.0% | 0.0% | 0.0% | 71.4% | 28.6% | 0.0% |  |
|  | Restorative dentistry | N | 4 | 2 | 1 | 5 | 36 | 21 | 1 |  |
|  |  | % | 5.7% | 2.9% | 1.4% | 7.1% | 51.4% | 30.0% | 1.4% |  |
| University | Private | N | 14 | 7 | 2 | 6 | 10 | 77 | 68 | 0.209 |
|  |  | % | 7.6% | 3.8% | 1.1% | 3.3% | 5.4% | 41.8% | 37.0% |  |
|  | Public | N | 15 | 11 | 5 | 9 | 3 | 103 | 66 |  |
|  |  | % | 7.1% | 5.2% | 2.4% | 4.2% | 1.4% | 48.6% | 31.1% |  |

**Appendix Table S2. Comparison of tools to detect a carious lesion across specialty and university**

|  | | Graduation university | | | | P-value | Years since graduation | | | | | | | | P-value | Postgraduate study | | | | P-value |
| --- | --- | --- | --- | --- | --- | --- | --- | --- | --- | --- | --- | --- | --- | --- | --- | --- | --- | --- | --- | --- |
|  |  | *Public University* | | *Private University* | |  | *Less than 5* | | *5 to 9* | | *10 to 14* | | *15 ≤* | |  | *Yes* | | *No* | |  |
|  |  | **N** | **%** | **N** | **%** |  | **N** | **%** | **N** | **%** | **N** | **%** | **N** | **%** |  | **N** | **%** | **N** | **%** |  |
| Do you believe there has been a significant change in the operative dental practice since your graduation year? | No | 18 | 6.4% | 7 | 6.2% | 0.270 | 20 | 10.7% | 4 | 5.4% | 1 | 1.2% | 0 | 0.0% | **<0.001** | 23 | 8.9% | 2 | 1.4% | **<0.001** |
|  | Maybe | 37 | 13.1% | 22 | 19.5% |  | 50 | 26.7% | 4 | 5.4% | 2 | 2.4% | 3 | 5.9% |  | 54 | 20.9% | 5 | 3.6% |  |
|  | Yes | 228 | 80.6% | 84 | 74.3% |  | 117 | 62.6% | 66 | 89.2% | 81 | 96.4% | 48 | 94.1% |  | 181 | 70.2% | 131 | 94.9% |  |
| I find it hard to get a trusted source for evidence-based information | Strongly agree | 28 | 9.9% | 12 | 10.6% | 0.805 | 25 | 13.4% | 6 | 8.1% | 6 | 7.1% | 3 | 5.9% | 0.363 | 30 | 11.6% | 10 | 7.2% | 0.158 |
|  | Agree | 114 | 40.3% | 52 | 46.0% |  | 80 | 42.8% | 32 | 43.2% | 39 | 46.4% | 15 | 29.4% |  | 112 | 43.4% | 54 | 39.1% |  |
|  | Unsure | 49 | 17.3% | 19 | 16.8% |  | 29 | 15.5% | 13 | 17.6% | 17 | 20.2% | 9 | 17.6% |  | 43 | 16.7% | 25 | 18.1% |  |
|  | Disagree | 86 | 30.4% | 28 | 24.8% |  | 49 | 26.2% | 21 | 28.4% | 21 | 25.0% | 23 | 45.1% |  | 66 | 25.6% | 48 | 34.8% |  |
|  | Strongly disagree | 6 | 2.1% | 2 | 1.8% |  | 4 | 2.1% | 2 | 2.7% | 1 | 1.2% | 1 | 2.0% |  | 7 | 2.7% | 1 | 0.7% |  |
| I find it hard to understand the results of scientific articles due to the statistical portion | Strongly agree | 28 | 9.9% | 12 | 10.6% | 0.366 | 24 | 12.8% | 5 | 6.8% | 9 | 10.7% | 2 | 3.9% | 0.271 | 33 | 12.8% | 7 | 5.1% | **<0.001** |
|  | Agree | 107 | 37.8% | 47 | 41.6% |  | 75 | 40.1% | 35 | 47.3% | 26 | 31.0% | 18 | 35.3% |  | 112 | 43.4% | 42 | 30.4% |  |
|  | Unsure | 41 | 14.5% | 21 | 18.6% |  | 30 | 16.0% | 9 | 12.2% | 16 | 19.0% | 7 | 13.7% |  | 41 | 15.9% | 21 | 15.2% |  |
|  | Disagree | 105 | 37.1% | 31 | 27.4% |  | 57 | 30.5% | 24 | 32.4% | 31 | 36.9% | 24 | 47.1% |  | 71 | 27.5% | 65 | 47.1% |  |
|  | Strongly disagree | 2 | 0.7% | 2 | 1.8% |  | 1 | 0.5% | 1 | 1.4% | 2 | 2.4% | 0 | 0.0% |  | 1 | 0.4% | 3 | 2.2% |  |
| I find it hard to understand the guidelines and consensus | Strongly agree | 9 | 3.2% | 4 | 3.5% | 0.432 | 8 | 4.3% | 3 | 4.1% | 1 | 1.2% | 1 | 2.0% | 0.769 | 10 | 3.9% | 3 | 2.2% | 0.054 |
|  | Agree | 57 | 20.1% | 28 | 24.8% |  | 44 | 23.5% | 16 | 21.6% | 15 | 17.9% | 10 | 19.6% |  | 60 | 23.3% | 25 | 18.1% |  |
|  | Unsure | 65 | 23.0% | 32 | 28.3% |  | 48 | 25.7% | 17 | 23.0% | 19 | 22.6% | 13 | 25.5% |  | 69 | 26.7% | 28 | 20.3% |  |
|  | Disagree | 135 | 47.7% | 45 | 39.8% |  | 81 | 43.3% | 34 | 45.9% | 41 | 48.8% | 24 | 47.1% |  | 110 | 42.6% | 70 | 50.7% |  |
|  | Strongly disagree | 17 | 6.0% | 4 | 3.5% |  | 6 | 3.2% | 4 | 5.4% | 8 | 9.5% | 3 | 5.9% |  | 9 | 3.5% | 12 | 8.7% |  |
| I feel some evidence-based information is time-consuming and not clinically applicable in my daily practice | Strongly agree | 29 | 10.2% | 10 | 8.8% | 0.894 | 24 | 12.8% | 3 | 4.1% | 8 | 9.5% | 4 | 7.8% | 0.073 | 28 | 10.9% | 11 | 8.0% | **0.001** |
|  | Agree | 111 | 39.2% | 46 | 40.7% |  | 85 | 45.5% | 30 | 40.5% | 27 | 32.1% | 15 | 29.4% |  | 117 | 45.3% | 40 | 29.0% |  |
|  | Unsure | 60 | 21.2% | 20 | 17.7% |  | 32 | 17.1% | 18 | 24.3% | 17 | 20.2% | 13 | 25.5% |  | 47 | 18.2% | 33 | 23.9% |  |
|  | Disagree | 70 | 24.7% | 32 | 28.3% |  | 42 | 22.5% | 20 | 27.0% | 24 | 28.6% | 16 | 31.4% |  | 60 | 23.3% | 42 | 30.4% |  |
|  | Strongly disagree | 13 | 4.6% | 5 | 4.4% |  | 4 | 2.1% | 3 | 4.1% | 8 | 9.5% | 3 | 5.9% |  | 6 | 2.3% | 12 | 8.7% |  |
| I feel the evidence-based information is not applicable in daily practice due to the economic burden | Strongly agree | 38 | 13.4% | 23 | 20.4% | 0.503 | 30 | 16.0% | 13 | 17.6% | 10 | 11.9% | 8 | 15.7% | 0.277 | 43 | 16.7% | 18 | 13.0% | 0.065 |
|  | Agree | 128 | 45.2% | 49 | 43.4% |  | 92 | 49.2% | 31 | 41.9% | 36 | 42.9% | 18 | 35.3% |  | 122 | 47.3% | 55 | 39.9% |  |
|  | Unsure | 56 | 19.8% | 19 | 16.8% |  | 36 | 19.3% | 12 | 16.2% | 14 | 16.7% | 13 | 25.5% |  | 48 | 18.6% | 27 | 19.6% |  |
|  | Disagree | 50 | 17.7% | 19 | 16.8% |  | 27 | 14.4% | 15 | 20.3% | 17 | 20.2% | 10 | 19.6% |  | 40 | 15.5% | 29 | 21.0% |  |
|  | Strongly disagree | 11 | 3.9% | 3 | 2.7% |  | 2 | 1.1% | 3 | 4.1% | 7 | 8.3% | 2 | 3.9% |  | 5 | 1.9% | 9 | 6.5% |  |

**Appendix Table S3. Comparison of Evidence-based practice across university of graduation, years since graduation, and postgraduate study**
