# Supplementary material for: Knowledge and attitudes toward evidence-based cariology and restorative dentistry among Egyptian dental practitioners: a cross-sectional survey
Source: BMC Oral Health. 2023 Sep 1;23:622. doi: 10.1186/s12903-023-03333-z (PMC10474780; doi:10.1186/s12903-023-03333-z)
Supplement: Supplementary file 2 — Supplementary Material 2 [file 12903_2023_3333_MOESM2_ESM.docx]

**Attitudes of Egyptian dental practitioners toward evidence-based cariology and restorative dentistry**

**Introduction:**

Thank you for showing an interest in this project!
The purpose of this study is to explore the Egyptian dental practitioners' perceptions and knowledge of evidence-based cariology and restorative dentistry.

Please respond to an online questionnaire in the next sections.

**Consent Form:**

I realize that this data is being gathered solely for research purposes, my participation in the project is entirely voluntary, and I am free to request additional information at any time.

**Personal information:**

1. What is your Profession?
2. What is your gender?
3. University
4. What is your specialty?
5. What is your highest degree?
6. University
7. Graduation year of highest degree
8. Primary sector of practice
9. Location of practice

**Caries management:**

1. What is your preferred caries classification for application in your dental practice? (Mention please)
2. What tools do you use to detect a carious lesion? (Mention please)
3. Please respond to the following statements:

|  | **Strongly agree** | **Agree** | **Unsure** | **Disagree** | **Strongly disagree** |
| --- | --- | --- | --- | --- | --- |
| 1. Caries care, Caries management, and Caries control all have the same meaning |  |  |  |  |  |
| 1. A cavity-free patient indicates that he/she is caries-free |  |  |  |  |  |
| 1. Management of caries lesion depends on its activity |  |  |  |  |  |
| 1. The risk assessment of the patient influences the management of caries |  |  |  |  |  |
| 1. The new approach of caries management depends on the ability of dental tissue remineralization |  |  |  |  |  |
| 1. The new concept of caries management is based on the sealing of cavities by altering the ecological niche of cariogenic bacteria |  |  |  |  |  |
| 1. The non-invasive approach is only indicated for patients with a high caries risk |  |  |  |  |  |
| 1. Caries diagnosis depends on dentin stains and cavitation |  |  |  |  |  |
| 1. The step-wise caries excavation is the best treatment for a deep lesion |  |  |  |  |  |
| 1. Step-wise caries removal is a type of selective caries removal |  |  |  |  |  |
| 1. It is necessary to remove soft dentin from the whale cavity |  |  |  |  |  |
| 1. Complete caries removal to hard dentin is the best treatment option for small to moderate-sized cavities |  |  |  |  |  |
| 1. Dentin hardness and color are the most critical features of dentin during cavity preparation |  |  |  |  |  |
| 1. In case of a cavitated lesion, a minimally invasive approach could be an alternative treatment |  |  |  |  |  |
| 1. Resistance to excavation is a characteristic sign during caries management |  |  |  |  |  |
| 1. The history of pain is critical in the caries management |  |  |  |  |  |
| 1. Routine x-rays on every patient are essential for caries detection |  |  |  |  |  |
| 1. Periapical radiographs are more effective than Bitewing in caries diagnosis |  |  |  |  |  |
| 1. Pulp testing may not be essential during caries management |  |  |  |  |  |
| 1. The explorer probe is necessary for caries diagnosis |  |  |  |  |  |
| 1. ICDAS classification is more accurate than Si/Sta classification |  |  |  |  |  |
| 1. The cavity sealing approach is only used on the intact enamel surface |  |  |  |  |  |
| 1. Sealing of the cavity could remineralize it |  |  |  |  |  |
| 1. It is necessary to seal inactive root caries |  |  |  |  |  |

**Continuous dental education:**

1. Do you believe there has been a significant change in the operative dental practice since your graduation year? (Yes/No)
2. What is your preferred method of staying up-to-date with cariology and restorative dentistry?

- University programs and degrees
- In-person courses and workshops
- Online courses
- Conferences
- Journal articles
- Textbooks
- Others

1. How many continuing education courses in cariology and restorative dentistry did you take in the last year?
2. How many international scientific articles on cariology, and restorative dentistry did you read in the last year?
3. What do you feel are the barriers to updating your cariology and restorative dentistry knowledge (Please select all which apply)?

- Courses for continuous education are expensive.
- The newly gained information will not be clinically applicable due to lack of equipment or working in the low-economic area.
- I think there has been no significant development in the field since my graduating year.
- Others

1. Do you always follow the recent consensus and guidelines in cariology and restorative dentistry? (Yes/No)
2. If yes, whose organizations or committees do you adhere to their consensus and guidelines?

**Evidence-based dentistry:**

1. In your opinion, what is the main challenging aspect of performing evidence-based cariology and restorative dentistry?
2. Please respond to the following statements:

|  | **Strongly agree** | **Agree** | **Unsure** | **Disagree** | **Strongly disagree** |
| --- | --- | --- | --- | --- | --- |
| 1. I find it hard to get a trusted source for evidence-based information |  |  |  |  |  |
| 1. I find it hard to understand the results of scientific articles due to the statistical portion |  |  |  |  |  |
| 1. I find it hard to understand the guidelines and consensus |  |  |  |  |  |
| 1. I feel some evidence-based information is time-consuming and not clinically applicable in my daily practice |  |  |  |  |  |
| 1. I feel the evidence-based information is not applicable in daily practice due to the economic burden |  |  |  |  |  |
